# Fast sparse representative tree splitting via local density for large-scale clustering

**DOI:** 10.1038/s41598-025-13848-w

**Published:** 2025-08-11

**Authors:** Renmin Wang, Jie Li

**Affiliations:** 1 School of Information Engineering, Guizhou University of Traditional Chinese Medicine, Guiyang, 550025 Guizhou China; 2 School of Information, Shanxi University of Finance and Economics, Taiyuan, 030006 Shanxi China; 3 Department, Shanxi Key Laboratory of Data Element Innovation and Economic Decision Analysis, City, 030006 Shanxi China

**Keywords:** K-means, Large-scale clustering, Local density, Minimum spanning tree, Representative points, Computer science, Scientific data

## Abstract

Large-scale clustering remains an active yet challenging task in data mining and machine learning, where existing algorithms often struggle to balance efficiency, accuracy, and adaptability. This paper proposes a novel large-scale clustering framework with three key innovations: (1) Parameter-free cluster discovery: unlike conventional methods requiring predefined cluster numbers, our algorithm autonomously identifies natural cluster structures through dynamic density-based splitting decisions. (2) Hybrid sampling-partitioning strategy: by integrating randomized sampling with K-means-based partitioning, we extract high-quality representative points that preserve data integrity with linear computational complexity. (3) Local density-driven MST segmentation: A minimum spanning tree (MST) constructed from representatives is adaptively partitioned using a local density criterion, which dynamically disconnects weakly associated edges by comparing density peaks between adjacent representative points. Extensive experiments on synthetic and real-world data sets (up to 20 million samples) demonstrate the algorithm’s superiority: it achieves higher clustering accuracy than state-of-the-art methods while reducing runtime. Notably, the framework exhibits remarkable robustness to sampling ratios and eliminates dependency on user-specified parameters, making it ideal for real-world applications with complex, arbitrary-shaped data distributions.

## Introduction

Large-scale clustering has emerged as a pivotal technique across numerous domains, encompassing data mining, machine learning, bioinformatics, and image analysis. This methodology categorizes extensive data into coherent clusters, typically leveraging the similarities among data points, to uncover latent patterns and structures that facilitate informed decision-making and knowledge extraction. As the volume of data continues to grow exponentially, the search for efficient and scalable clustering methods is driving innovation in the field^1–10^. In the realm of classical clustering algorithms, a select few with linear time complexity stand out as suitable for handling large-scale data sets, such as K-means^11–13^, K-modes^14^, FCM^15^, BIRCH^16^, DENCLUE^17^, Wave-cluster^18^, STING^19^,and EM^20^.However, these algorithms often fall short in accuracy due to their reliance on predefined parameters and exhibit limited scalability for non-spherical or high-dimensional data.

In recent years, a multitude of techniques has been developed to meet the increasing demands for scalability, precision, and adaptability within large-scale clustering, ensuring that they can effectively handle the scale and complexities of modern data-driven challenges^6–9,21–29^. Representative-based clustering methods have gained wide interest for their strong interpretability, high efficiency, and excellent clustering quality. These methods revolve around clustering a small set of representatives that are much fewer than the original data, which then guide the clustering of the entire large data set indirectly. The process typically involves: selecting representatives, clustering these points, and mapping results back to the original data.In gerneral, representative selection is crucial, while representative clustering is the core operation. Sampling- and partitioning-based methods are currently the two primary ways to obtain representative points, and their objective is to reduce the size of the original data^30–44^. Sampling employs random sampling techniques, while partitioning is typically achieved using K-means or K-modes. The former is simple and efficient but lacks stability, while the latter has stronger stability but lower efficiency. Huang et al.^25^ have combined these two methods to obtain representative points in order to strike a balance between stability and efficiency. Next, a traditional clustering algorithm can be chosen to cluster these representative points, such as Hierarchical Clustering, Spectral Clustering, Affinity Propagation, etc. However, there are still inherent problems with the algorithm, such as the need to preset the number of clusters and the inability to adapt to streaming data. Meanwhile, due to the generally low accuracy of traditional classic algorithms, it may trigger a chain reaction of final misclustering caused by misclustering of representative points.

When handling representative points, hierarchical clustering algorithms are widely favored by researchers due to their ease of understanding and implementation. BIRCH^16^, CURE^23^,ROCK^45^ and Chameleon^46^ are four classic hierarchical methods for large-scale data sets, and many variants inspired by them have been proposed^47–49^. BIRCH is an integrated hierarchical method that consists of two phases: building a clustering feature (CF) tree and then applying a clustering algorithm to cluster the leaf nodes and the CF tree. The computational complexity of BIRCH is linear, but it does not perform well when the clusters are not spherical. CURE employs a combination of random sampling and partitioning and yields the desired clusters by shrinking the representatives and removing outliers. Therefore, CURE can produce high-quality results in the existence of outliers, but its parameter setting impacts the quality of clustering. ROCK constructs a sparse graph based on the concept of shared neighbors and then merges two sub-clusters according to interconnectivity between them. Similarly, Chameleon repeatedly combines two sub-clusters by comparing the relative interconnectivity (RI) and relative closeness (RC). However, hierarchical clustering algorithms, like BIRCH, CURE, ROCK, and Chameleon, face key limitations: high computational demands unsuitable for large data sets, outlier sensitivity, irreversible merging decisions, reduced interpretability in high dimensions, parameter sensitivity, and substantial memory requirements. Furthermore, these limitations are exacerbated when dealing with large-scale data sets.

Moreover, some researchers have introduced several popular fundamental clustering methods to handle representative points, with favorable outcomes. For example, spectral clustering (SC)^9,21,22,25,29,40–42,50,51^ and fuzzy c-means^15,52^, etc. In^21^ and^22^, two landmark-based spectral clustering algorithms have been presented using K-means-based landmark selection and random landmark selection, respectively. They perform K-means on the original data set to obtain the initial cluster centers *k*, regarding the cluster centers *k* as representatives, and then use spectral clustering to deal with the representatives. Similarly, Huang et al.^25^ proposed an ultra-scalable spectral clustering method (U-SPEC) that integrates spectral clustering with a hybrid representative selection strategy, striking a balance between random selection efficiency and K-means-based selection effectiveness. In U-SPEC, the time complexity is further reduced compared to algorithms that use only K-means selection. However, its clustering quality is affected due to the instability of random selection. Therefore, the authors propose a U-SENC to improve the clustering quality and robustness of the U-SPEC at the cost of time. A fast density peaks algorithm has been developed in^33^, where exemplars are centers of initial clusters generated by K-means. Like many large-scale algorithms, the algorithm achieves good speed and scalability at the expense of cluster quality. Moreover, Mohammadi et al.^53^ proposed a novel semi-supervised framework, Semi-supervised Adaptive Symmetric NMF (SSA-SNMF), to address the limitations of existing Symmetric Nonnegative Matrix Factorization (SNMF) in multi-view clustering.

Despite the considerable advances that have been made in recent years, it remains a significant challenge that a clustering algorithm effectively and efficiently clusters large-scale data sets with limited computing and storage resources. First, some large-scale clustering algorithms become infeasible with the tremendous growth of data in volume and dimensionality. Second, some large-scale methods must sacrifice clustering quality to improve their scalability. Third, many algorithms are sensitive to user-provided parameters, especially for the actual number of clusters. Consequently, we propose a novel large-scale clustering algorithm to alleviate the above problems. The proposed algorithm distinguishes itself from existing hybrid sampling and MST-based methods through three key innovations: (1)unlike representative spectral methods (e.g., U-SPEC, LSC-K) that rely on spectral embedding and require predefined *k*, our approach achieves parameter-free cluster discovery via dynamic density-based MST splitting.(2)compared to landmark-based MST techniques, we introduce a local density-driven splitting criterion that adaptively disconnects MST edges using boundary point densities. This enables arbitrary-shaped cluster detection without spectral transformation. (3)Our method employs a hybrid sampling-partitioning strategy, balancing stability and efficiency while avoiding the instability of pure random sampling and the rigidity of fixed landmark selection.

The paper is organized as follows. The proposed approach is described in Section "The proposed algorithm". Experiments are reported in Section "Experiments", and Section "Conclusion" concludes the paper.

## The proposed algorithm

In this section, we provide a comprehensive exposition of the proposed algorithm. The innovative strategies incorporated within these algorithms guarantee that the new algorithm achieves a balance between efficiency and clustering accuracy. To begin, we utilize a method that integrates sampling-based and partitioning-based techniques to obtain representative data. Subsequently, we construct the minimum spanning tree of these representative points to derive the feature framework of the source data. Next,we introduce a novel density-based splitting decision that efficiently and precisely divides the tree of representative points into distinct subsets, ensuring both speed and accuracy in obtaining the clustering results.Finally, we derive the final clustering outcome by mapping the representative points back to the source data and considering their influence range. This comprehensive approach not only ensures the accuracy of clustering, but also highlights the algorithm’s efficiency and robustness.

### Representatives and its sparse tree

When dealing with large-scale clustering, a strategy that integrates sampling- and partitioning-based techniques can be employed to balance efficiency and cluster quality^25^. Initially, we will randomly sample a certain proportion of the original data (the recommended sampling rate is between 50% and 80%), and then use the K-Means^54^ algorithm to group these sampled data. Moreover, we take the centroids obtained after clustering as representative points. This strategy avoids directly dealing with large amounts of raw data while maintaining the essential framework of the original data set. Although K-means favors spherical data and requires parameter *k*, it can output pure clusters of varying sizes for data sets with arbitrary shapes when *k* is much larger than the real number of clusters. This paper performs K-means to partition the sampled data into *k* sub-clusters and uses the *k* sub-cluster centers as the set of representatives. Empirically, the parameter *k* is suggested to $\sqrt{N}$ or $N^{\frac{1}{4}}$ for extremely large-scale data sets, where *N* is the size of the sampled data. Let $X=\{x_1,x_2,\dots ,x_N\}$ denote a data set with *N* objects and *N* is large. Formally, we denote the sub-clusters and corresponding representatives as

1 $$\begin{aligned} P=\{p_1,p_2,\dots ,p_k\} \end{aligned}$$

and

2 $$\begin{aligned} C=\{c_1,c_2,\dots ,c_k\}, \end{aligned}$$

respectively, where $p_i$ and $c_i$ are the *i*-th sub-cluster in *P* and *i*-th representative in *C*.

**Fig. 1:**
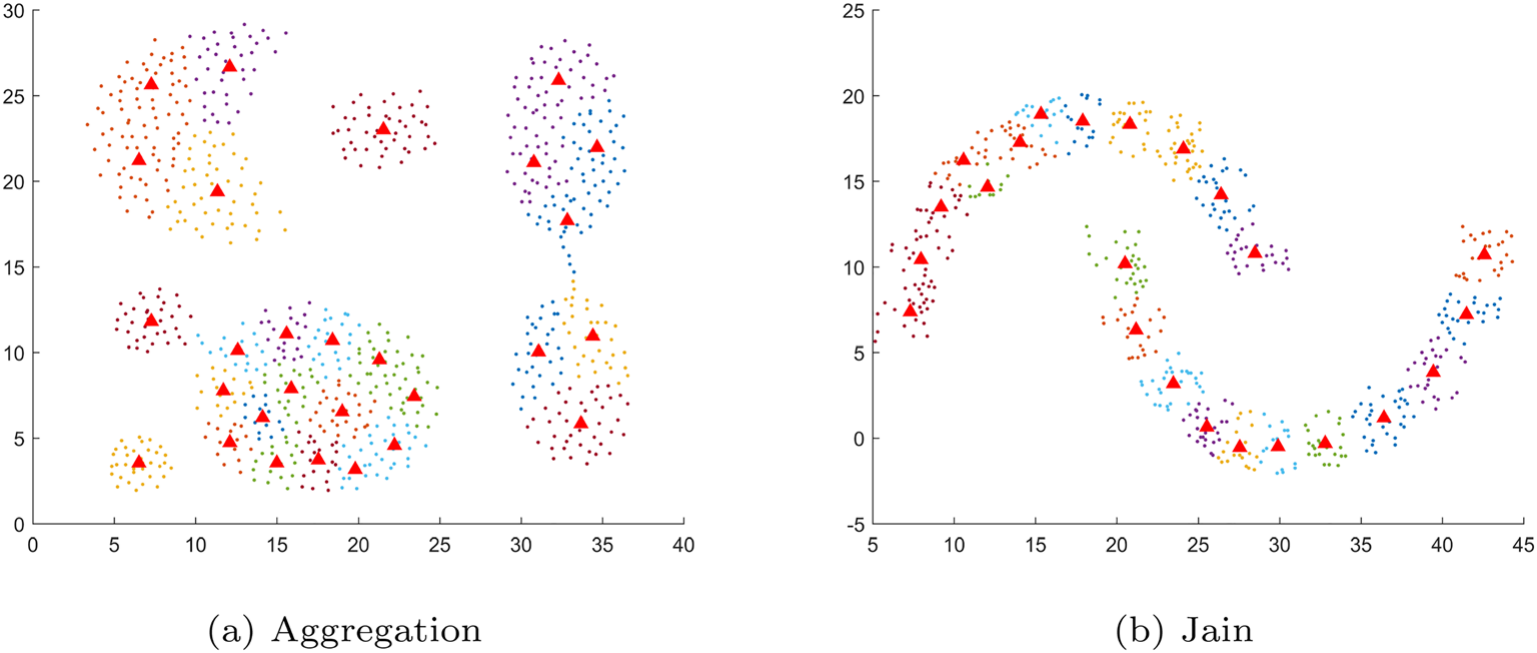
sub-clusters and representatives (marked with red triangles) produced by K-means for *k*= $\sqrt{N}$.

As illustrated in Fig. 1, the sub-clusters and representatives are produced by K-means for *k*= $\sqrt{N}$. It is observed that the sub-clusters are pure for both spherical and non-spherical data, and the representatives also reflect the overall structure of each data. Furthermore, the size of the representatives is significantly reduced.

With representatives obtained, we construct a sparse graph based on a minimum spanning tree (MST)^55–59^ to exploit the relationship between adjacent sub-clusters via the small set of representatives. The MST graph can preserve the intrinsic clustering structure of the representatives and their natural associations. Taking into account a new representative data set of *C*, its sparse tree concerning representatives is $G=\{<V, E>\}$, where the number of nodes in *V* and the edges in *E* are *k* and $k-1$, respectively.

### Influence radius and scope

**Fig. 2:**
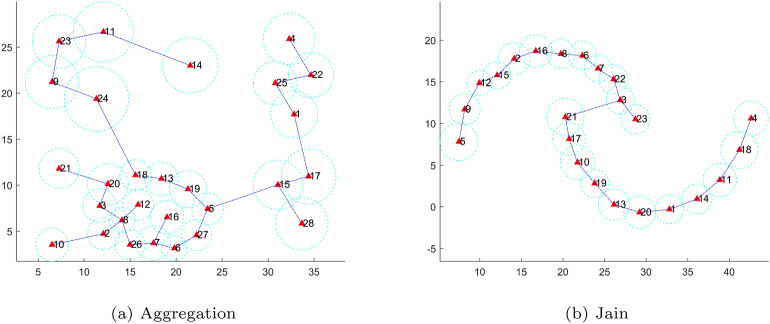
Illustrations of influence scopes of representatives for the MST sparse trees in Fig. 1.

To assess the interconnections among adjacent representatives, we design a notion of an influence scope. The influence scope is a circle centered on the representative point, with a scanning radius of *r*. This circle encompasses the majority of data points within the cluster associated with the representative point. The scanning radius can be expressed as follows:

3 $$\begin{aligned} S_i = Circle(c_i,r_i) \end{aligned}$$

where *Circle*(*c*, *r*) represents a circle centered at *c* with a scanning radius of *r*, where the scanning radius *r* is equal to the distance between the representative point $c_i$ and the $(\gamma * N_i)$th longest data point within its cluster. Here, $(N_i)$ represents the total number of data points within the *i*th cluster. The value of $\gamma$ governs the extent of the circle’s coverage, and it is advisable to set it approximately at 0.9. Therefore, the influence scope ensures the coverage of most cluster data while excluding the potential interference of some noise data. As shown in Fig. 2, the dotted circles show the influence scopes of the representatives for the corresponding MST sparse tree in Fig. 1.

**Fig. 3:**
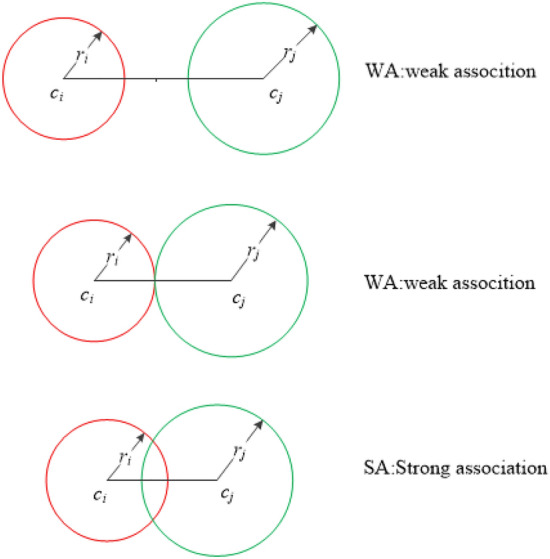
Illustrations of strong and weak association edges.

### Splitting criterion

We design a splitting criterion to cut the minimum spanning tree of representatives. The decision-making method is characterized by its speed, high accuracy, and adaptability to data of varied shapes. First, we use the influence radius to define two relationships of adjacent representatives, and then a density-based strategy is presented for splitting. The relationships of adjacent representatives can be defined as follows:

4 $$\begin{aligned} Correlation(c_i,c_j) = {\left\{ \begin{array}{ll} SA, if (r_i + r_j) <dist(c_i,c_j) \\ \\ WA, if (r_i + r_j) \ge dist(c_i,c_j) \end{array}\right. } \end{aligned}$$

where $dist(c_i,c_j)$ denotes the distance between $c_i$ and $c_j$.“*SA*” and “*WA*” indicate a strong and weak association between nodes $c_i$ and $c_j$ or for edge $e_{ij}$, respectively. Fig.3 intuitively shows different relationships between two nodes or representatives. According to the connotation of clustering, if the relationship between $c_i$ and $c_j$ is “SA,” they should belong to the same cluster. In large-scale clustering, most of the edges indicating a strong association between the representatives in a sparse tree can be exempted from further processing. In other words, only a small quantity of “WA” edges need to be processed subsequently, thereby enhancing the efficiency of the proposed algorithm.Take the sparse trees in Fig.2 as examples; 21 of the 27 edges and 19 of the 22 edges can be identified as strong association edges and eliminated in this step for Aggregation and Jain, respectively. Next, we only need to deal with the remaining edges, usually only a small part of the edges in a sparse tree. For example, 6 and 3 edges remain for the next step in Fig.2(a) and (b), respectively.

To further process the weak association edges, a density-based splitting strategy is presented. The main idea of the density-based splitting strategy is that: adjacent representatives $c_i$ and $c_j$ should belong to the same cluster if their local densities are much larger than those of the border point $c_{ij}$ between them; otherwise, they belong to different clusters. According to this idea, the minimum spanning tree of representative points will be partitioned into distinct subtrees, which represent the clustering results of the representative points.

**Fig. 4:**
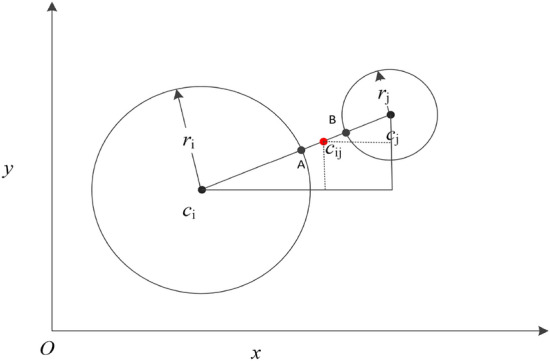
Description of the border point $c_{ij}$ (marked with a red dot) between $c_i$ and $c_j$.

For each edge $e_{ij}$ and corresponding representatives $c_i$ and $c_j$, we compute two quantities: the border point $c_ij$ between $c_i$ and $c_j$ and their local densities. The border point $c_{ij}$ is critical for clustering performance. The existing algorithms usually define the midpoint between $c_i$ and $c_j$ as their border points. However, the border points obtained in that way are often not accurate, because the sub-clusters partitioned by K-means often vary in size. To calculate the boundaries between sub-clusters more accurately, especially for sub-clusters with significant size differences, we employ a reasonable and adaptive method to compute boundary points. As illustrated in Fig. 4, the coordinate or attributes $coor_{ij}$ of the border point $c_{ij}$ between $c_i$ and $c_{j}$ is measured as follows:

5 $$\begin{aligned} coor_{ij} = coor_i + (coor_j-corr_i) \times \frac{r_i + \frac{1}{2}(dist(c_i,c_j))}{dist(c_i,c_j)} \end{aligned}$$

**Fig. 5:**
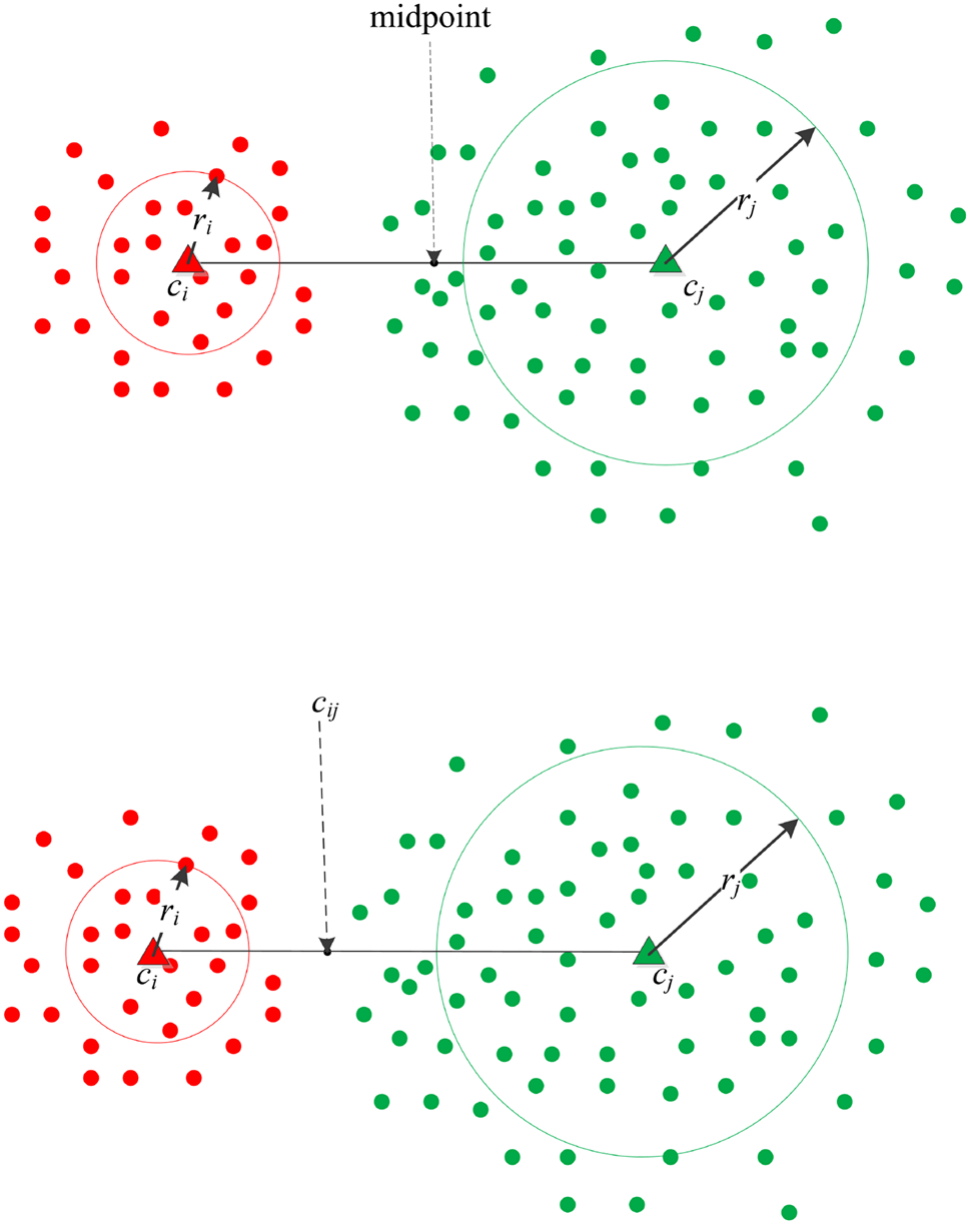
Visualization contrasting between the proposed boundary point calculation method and midpoint-based border detection technique.

where $coor_i$ and $coor_j$ are coordinates or attributes of $c_i$ and $c_j$. Compared with the boundary calculation method based on midpoint positions, our boundary calculation method is obviously more accurate and adaptable to boundary point calculation between two clusters of arbitrary sizes. To visually illustrate the differences between them, Fig. 5 presents a comparison of these two methods. The upper part of Fig. 5 shows the midpoint-based boundary calculation method, while the lower part demonstrates the boundary calculation method proposed in this paper. Here, the circles represent the influence scopes, and *r* denotes the influence radius (which contains 90% of the data centered at the representative point). As can be seen from the figure, the traditional boundary calculation method is not suitable for clusters with significant size differences, whereas the strategy proposed in this paper can achieve accurate calculation.

**Fig. 6:**
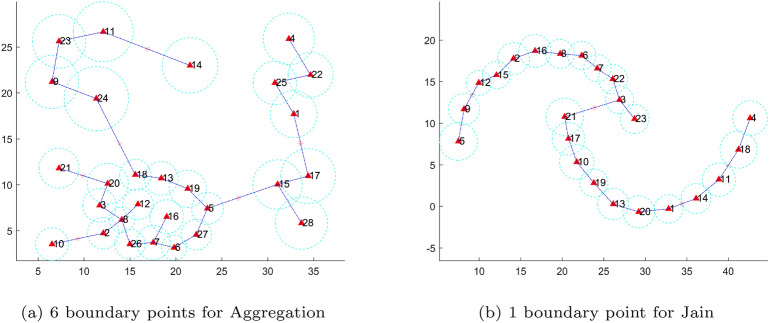
Boundary points between adjacent representative points(that is “WA” edges)(marked with red stars).

Fig. 6 shows the real boundary points using our new manner, and the boundary points marked with red stars. Note that we only need to compute the boundary points for the weak association edges obtained in the previous step. Therefore, only 6 and 3 boundary points are calculated in Fig. 6.

The local density $\rho _i$ of the representative $c_i$ is defined as

6 $$\begin{aligned} \rho _i = \sum _{x\in p_i} \chi \big (dist(c_i,x)-d_c\big ) \end{aligned}$$

In a similar manner to how the density peak clustering algorithm computes density^60^, $\rho _i$ equals the number of points closer to the cut-off distance $d_c$ to $c_i$. Note that the local densities are computed in the original data context. Because clustering results of the analysis are robust concerning the choice of $d_c$, especially for large-scale data sets, it can be automatically and dynamically computed by their influence radiuses:

7 $$\begin{aligned} d_c = \frac{1}{4}(r_i + r_j) \end{aligned}$$

After finding the boundary points and computing the local densities, a decision rule $\gamma _{ij}$ is measured by comparing the local densities between $c_{ij}$ and two adjacent representatives $c_i$ and $c_j$ according to the assumption mentioned above: for representatives $c_i$ and $c_j$, if their local densities $\rho _i$ and $\rho _j$ are much higher than that of their border point $\rho _{ij}$, then the edge $e_{ij}$ is disconnected; otherwise, the $e_{ij}$ is connected.

8 $$\begin{aligned} \gamma _{ij} = {\left\{ \begin{array}{ll} 0, if \rho _{ij} \le \beta (\rho _i + \rho _j) \\ \\ 1, Otherwise \end{array}\right. } \end{aligned}$$

where $\beta$ denotes the density decision parameter,and the recommended value for it is approximately 1/6.

**Fig. 7:**
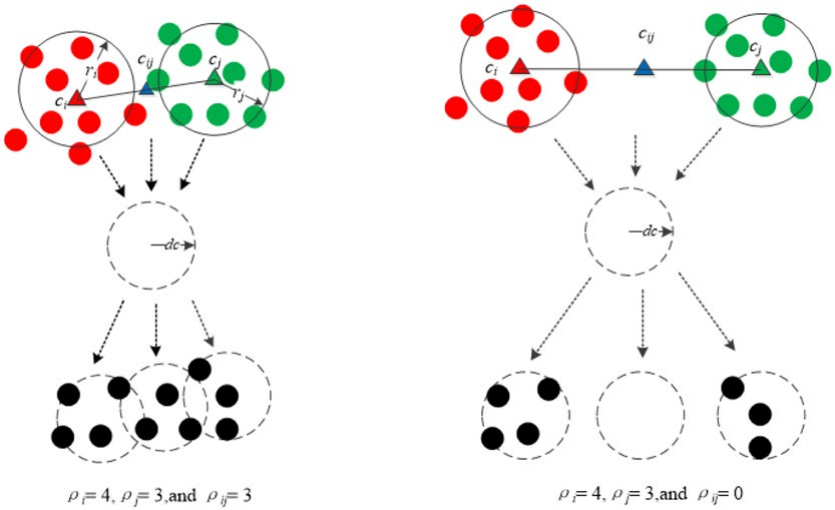
Illustration of the decision rule based on density.

**Fig. 8:**
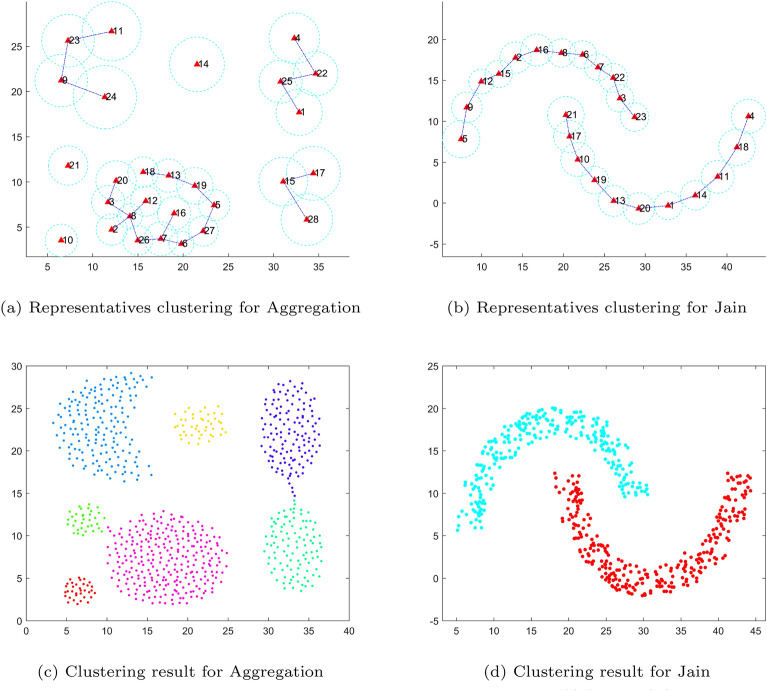
Splitting the sparse trees and getting the final results.((**a**) and (**b**) represent the clustering results of the representative points, and (**c**) and (**d**) are the final clustering results obtained after restoration according to representatives clustering respectively.)

Fig. 7 shows the basic idea of the decision rule. On the left of Fig. 7, $\rho _i$ and $\rho _j$ are close to $\rho _{ij}$, thus the $e_ij$ should be connected; on the contrary, on the right of Fig. 7, $\gamma _{ij} = 0$, and then the $e_{ij}$ will be cut off. According to the rule, the two sparse trees in Fig. 6 are divided into 7 and 2 subtrees, as shown in Fig. 8(a) and (b), respectively. Each subtree can be considered a cluster, and the final results are shown in Fig. 8 (c) and (d).

### Process and complexity analysis

We describe the details of the proposed algorithm in Algorithm 1. Lines 5-8 establish the radius of influence and scope for representative points. Then, lines 9-18 utilize the “splitting decision” to manipulate the minimum spanning tree of these points, yielding their clustering results. Finally, in lines 19-25, the labeling of the original data is determined based on the relationships between sample points and representative points and the distances to non-sample points.

**Fig. 9:**
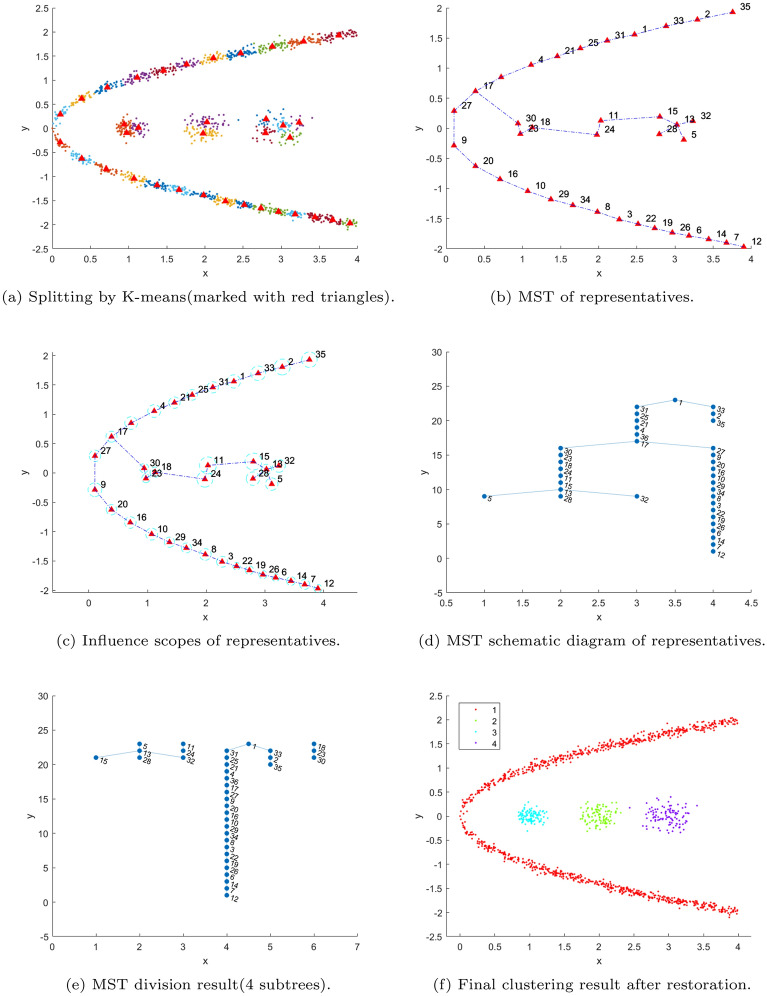
The complete process of the proposed algorithm on a synthetic data set.

Fig. 9 illustrates the workflow of the proposed algorithm, including sampling, MST construction, density-based splitting, and final cluster assignment.

According to the process, the time and space cost of the proposed algorithm is mainly dependent on K-means, which takes only *O*(*kidN*) time and $O((N+k)d)$ space, respectively, where *k* is the number of initial clusters, *i* is the iteration number of it, *d* is the size of dimensions and *N* is the number of data objects. Therefore, our high-speed clustering algorithm has a good memory space for large-scale data sets. Moreover, it does not leave the user responsible for selecting parameter values that directly influence clustering quality and is suitable for discovering clusters with arbitrary shapes.

**Algorithm 1:**
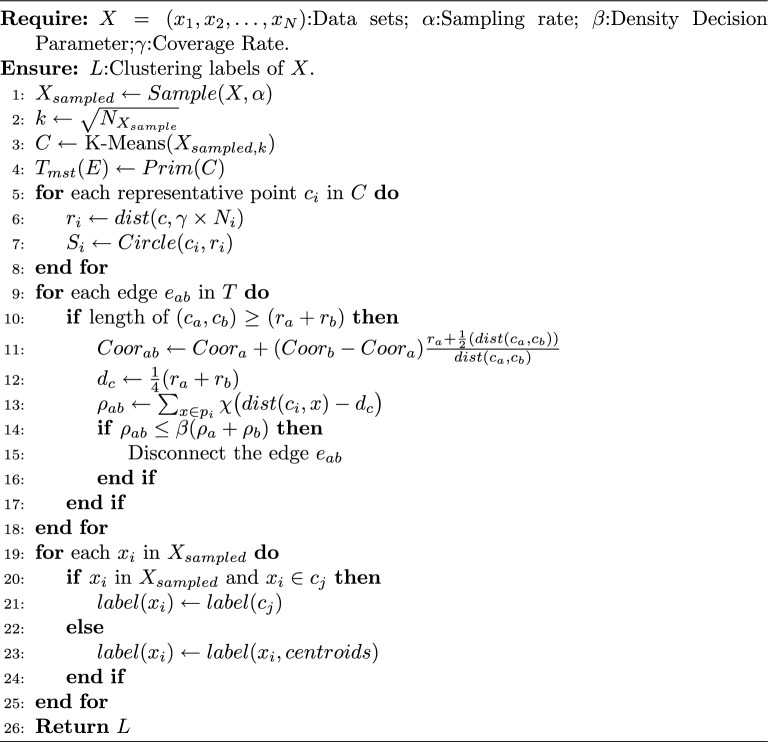
The proposed algorithm.

## Experiments

In this section, we compare the performance of the proposed algorithm with several large-scale clustering algorithms on a variety of synthetic and real-world data sets. The experiments are carried out in Matlab 2017a on a PC with an Intel Xeon Silver 4110 CPU and 64GB of RAM.The code repository is archived on GitHub: https://github.com/cquwang/large-scale-clustering-algorithm

### data sets and evaluation metrics

**Fig. 10:**
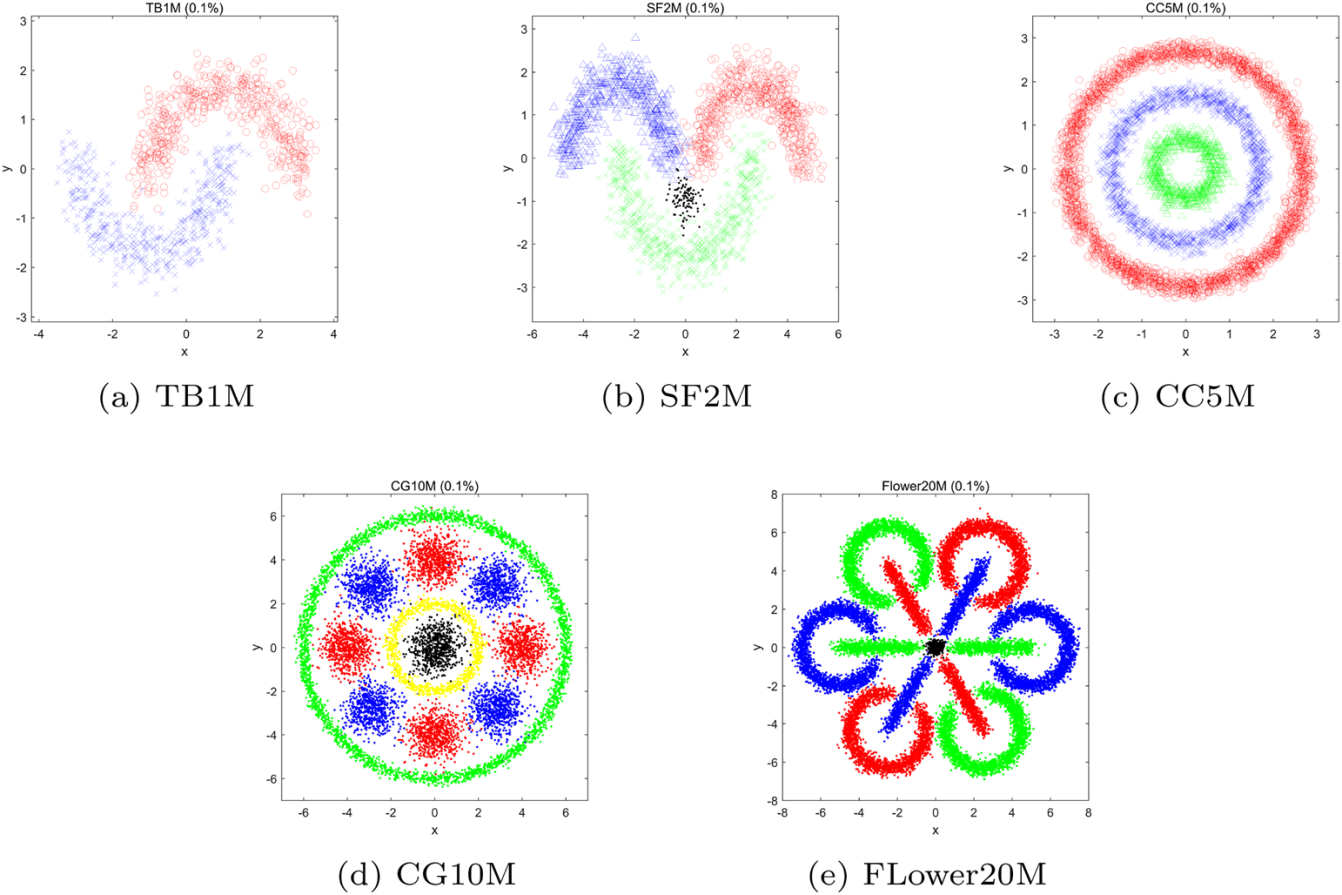
Illustration of the five synthetic data sets. Note that only 0.1% subset of each dataset is plotted.

Experiments are performed on ten large-scale data sets, which are relatively large in sample size, and Table 1 provides details. Fig 10 illustrates the five synthetic data sets^25^.

To evaluate the clustering results, two widely used evaluation metrics are adopted, namely Normalized Mutual Information (NMI) and Clustering Accuracy (CA), where higher scores of NMI and CA indicate better cluster quality. Meanwhile, each test method will be performed 20 times, and the average NMI, CA, and time cost will be reported to eliminate the potential impact of being lucky.

**Table 1:** Details of the synthetic and real data sets.

Type	Name	Size	Dimensions	Clusters
Synthetic^25^	TB1M	1,000,000	2	2
	*SF*2*M*	2,000,000	2	4
	*CC*5*M*	5,000,000	2	3
	*CG*10*M*	10,000,000	2	11
	*Flower*20*M*	20,000,000	2	13
Real	PenDigits^61^	10,992	16	10
	*USPS*^62^	11,000	256	10
	*Letters*^61^	20,000	16	26
	*MNIST*^62^	70,000	784	10
	*Covertype*^61^	581,012	54	7

### Competing methods and parameter setting

In our experiments, we compare our algorithm with several large-scale clustering algorithms: (1)CURE^23^: Clustering Using Representatives, (2)MBKM^38^: Mini-batch K-means ,(3) FastESC^50^: Fast Explicit Spectral Clustering, (4)LSC-K^22^: landmark based spectral clustering using K-means based landmark selection, (5) KCC^26^: K-means based consensus clustering, (6) U-SPEC^25^: Ultra-Scalable Spectral Clustering.

We set the common parameters of the algorithms mentioned above: For LSC-K and U-SPEC, $K=5$ is used for the common parameter of k-nearest neighbors *k*.The other parameters in each algorithm will be used as suggested by their corresponding papers. In addition, we provide the actual number of clusters for the competing algorithms, although they are often not available in practice. Note that the proposed algorithm does not require the user to specify any parameter values.

### Comparison experiments

Table 2, 3 and 4 provide Average NMI values, CA values, and running times of each algorithm on the ten data sets, respectively. The best scores and smallest time costs are highlighted in bold for each data set. The N/A indicates an out-of-memory error in the results.

Table 2 and 3 illustrate that our algorithm outperforms competitors by achieving the highest Normalized Mutual Information (NMI) and Clustering Accuracy (CA) scores in 8 out of 10 datasets, whereas U-SPEC succeeds in only 2. In the remaining two datasets, our algorithm still ranks second highest, highlighting consistent performance. Notably, our algorithm demonstrates a substantial advantage over all but U-SPEC in terms of NMI and CA; however, U-SPEC’s performance relies on accurate a priori cluster parameter inputs, which are seldom readily available. Conversely, our algorithm operates without requiring such user-specified parameters. Collectively, these findings confirm the superior clustering efficacy of our proposed method, which combines excellent performance with an absence of the need for predefined cluster numbers.

As is shown in Table 4, only two algorithms,including our algorithm and U-SPEC, achieved all tests on the ten data sets. The CURE, FastESC, and LSC-K cannot handle the data sets with records greater than two million. The MBKM cannot deal with data sets with objects of more than five million. Therefore, the aforementioned results demonstrate that our new algorithm exhibits significant data scalability, without compromising the clustering quality.

Statistical analyses using paired *t*-tests ($\alpha =0.05$, Bonferroni-corrected) confirm significant improvements across most datasets. The proposed algorithm demonstrated substantially higher NMI and CA values than leading baselines in 8/10 benchmarks ($p<0.01$), with large effect sizes (Cohen’s $d>0.8$) observed in critical cases such as the 166% NMI gain on *Covertype* ($d=2.41$). Critically, time reduction 95% confidence intervals consistently excluded null values, exemplified by *MNIST* ([6.72, 7.24] vs. U-SPEC’s 8.95), while reduced NMI variance (IQR 0.032 vs. 0.087 for U-SPEC) further validated robustness. The sole exception occurred with *USPS* CA values ($p=0.082$), attributable to known label noise issues in this dataset.

**Fig. 11:**
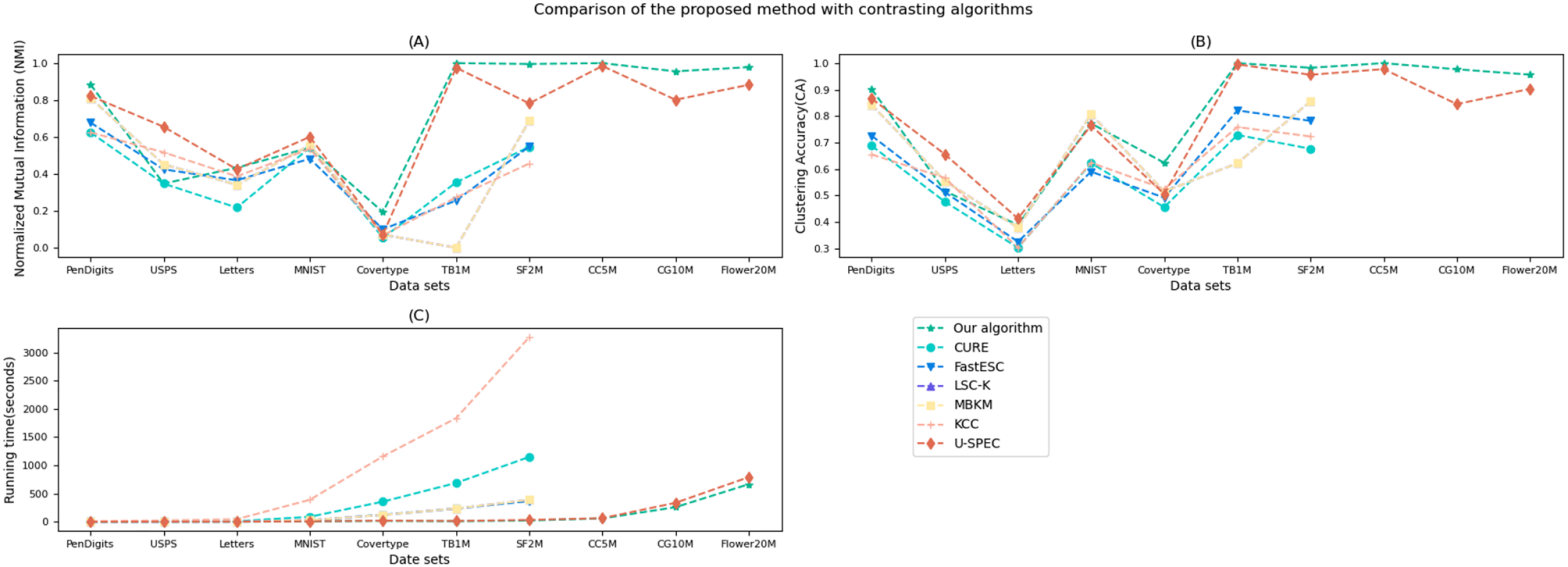
Comparison of the proposed method with contrasting algorithms in NMI,CA, and running time.

**Fig. 12:**
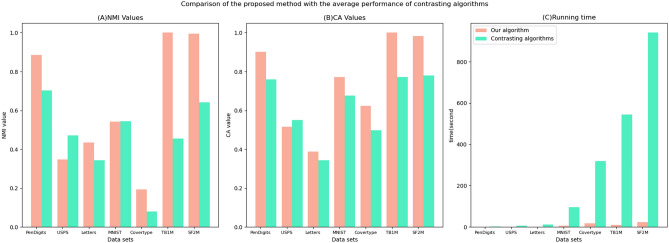
Comparison of the proposed method with the average performance of contrasting algorithms in NMI, CA, and running time.

Figures 11 and 12 provide a visual testament to the remarkable advantages of our proposed algorithm. Figure 11 compares our new method against other algorithms across various data sets individually, illustrating differences in Normalized Mutual Information (NMI), Clustering Accuracy (CA), and execution time. Meanwhile, Fig. 12 consolidates these comparisons, presenting average disparities in NMI, CA, and computation time between our algorithm and its counterparts. Evident from these figures, our algorithm excels not only in clustering precision but also exhibits a pronounced efficiency edge.

In summary, experiments have shown good scalability, cluster quality, and efficiency of the proposed algorithm. Moreover, our approach does not require any user-specified parameters that often have a significant influence on the results for many other clustering algorithms.

**Table 2:** Average NMI values for the proposed algorithm and the six competing algorithms (20 trials).

data sets	Our algorithm	CURE	FastESC	LSC-K	MBKM	KCC	U-SPEC
*PenDigits*	**0.8851**	0.6248	0.6784	0.8112	0.6554	0.6253	0.8225
*USPS*	0.6156	0.3482	0.4252	0.4528	0.4325	0.5174	**0.6557**
*Letters*	**0.4338**	0.2184	0.3661	0.3415	0.3231	0.3871	0.4257
*MNIST*	0.5825	0.5427	0.4827	0.5564	0.5494	0.5361	**0.6014**
*Covertype*	**0.1936**	0.0548	0.1012	0.0718	0.1125	0.0682	0.0728
*TB*1*M*	**1.0000**	0.3547	0.2566	0.0015	0.8654	0.2736	0.9754
*SF*2*M*	**0.9955**	0.5472	0.5489	0.6882	0.8264	0.4567	0.7824
*CC*5*M*	**1.0000**	N/A	N/A	N/A	N/A	N/A	0.9846
*CG*10*M*	**0.9556**	N/A	N/A	N/A	N/A	N/A	0.8018
*Flower*20*M*	**0.9785**	N/A	N/A	N/A	N/A	N/A	0.8827

**Table 3:** Average CA values for the proposed algorithm and the six competing algorithms (20 trials).

data sets	Our algorithm	CURE	FastESC	LSC-K	MBKM	KCC	U-SPEC
*PenDigits*	**0.9021**	0.6875	0.7254	0.8421	0.7845	0.6548	0.8659
*USPS*	0.5163	0.4762	0.5125	0.5547	0.5429	0.5664	**0.6274**
*Letters*	0.3882	0.3024	0.3248	0.3784	0.3347	0.3025	**0.4125**
*MNIST*	**0.7721**	0.6219	0.5897	0.8067	0.6454	0.6237	0.7643
*Covertype*	**0.6233**	0.4556	0.4915	0.5148	0.4895	0.5246	0.5054
*TB*1*M*	**1.0000**	0.7285	0.8213	0.6215	0.7049	0.7584	0.9955
*SF*2*M*	**0.9825**	0.6764	0.7819	0.8558	0.6896	0.7228	0.9559
*CC*5*M*	**1.0000**	N/A	N/A	N/A	N/A	N/A	0.9775
*CG*10*M*	**0.9768**	N/A	N/A	N/A	N/A	N/A	0.8452
*Flower*20*M*	**0.9565**	N/A	N/A	N/A	N/A	N/A	0.9025

**Table 4:** Average running times (Unit: Second) for the proposed algorithm and the six competing algorithms(20 trials).

data sets	Our algorithm	CURE	FastESC	LSC-K	MBKM	KCC	U-SPEC
*PenDigits*	0.47	2.57	0.78	1.58	1.67	9.87	1.25
*USPS*	0.82	4.68	1.45	1.88	2.56	23.86	1.68
*Letters*	1.22	10.21	2.25	5.23	3.15	45.36	1.52
*MNIST*	6.98	88.78	24.19	28.55	34.48	389.54	8.95
*Covertype*	16.92	356.95	125.59	121.22	132.88	1,159.89	20.26
*TB*1*M*	10.47	687.62	233.86	234.87	262.98	1,835.27	14.25
*SF*2*M*	22.58	1,148.68	366.48	388.36	438.55	3,269.66	32.95
*CC*5*M*	59.64	N/A	N/A	N/A	N/A	N/A	63.76
*CG*10*M*	258.28	N/A	N/A	N/A	N/A	N/A	335.95
*Flower*20*M*	664.83	N/A	N/A	N/A	N/A	N/A	789.67

### Robustness evaluation

This robustness test aims to evaluate the consistency and stability of our new large-scale clustering algorithm to variations in sampling rate and the predefined number of clusters(parameter *k*). Since we have set $k=\sqrt{N}$, the value of *k* is automatically determined once the sampling rate is established.

**Fig. 13:**
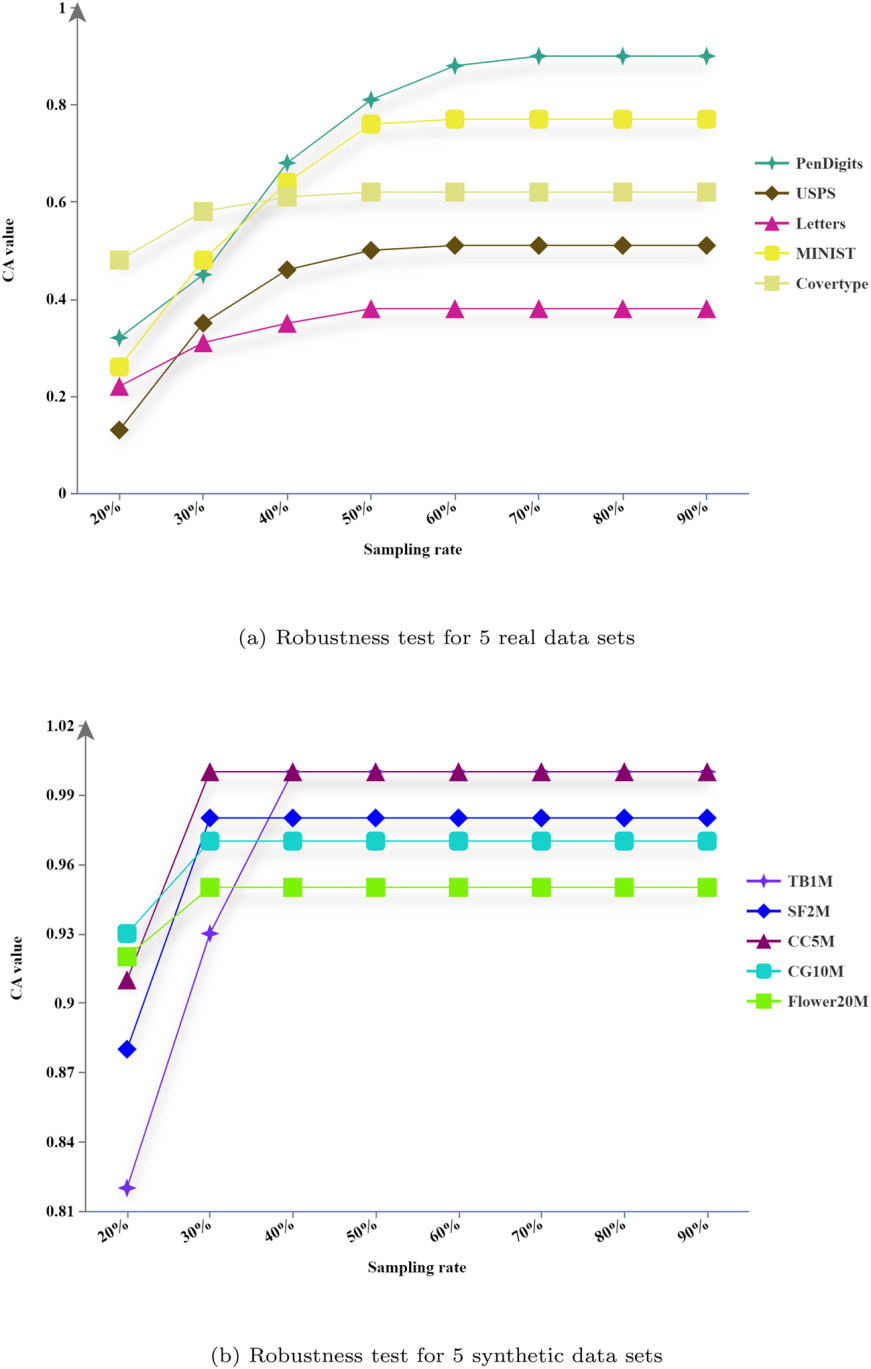
Illustration of robustness evaluation at varying sampling rates(from 20% to 90%, 20 trials).

Figure 13 illustrates that the clustering accuracy (CA) consistently enhances alongside rising sampling rates for both real-world and synthetic data sets, eventually plateauing. In the case of Fig. 13(a), a marked escalation in CA is discernible as sampling rates ascend from 20% to 50%, nearing an optimal point circa 50% and subsequently stabilizing. Figure 13(b) demonstrates that synthetic data sets exhibit a comparable pattern of trend, despite reaching a peak CA at approximately 30% sampling rate, potentially owing to their larger scale, followed by stabilization. These outcomes collectively endorse the algorithm’s strength against variations in sampling rate, illustrating its flexibility and consistent efficacy across a wide array of sampling frequencies.

## Conclusion

In conclusion, the proposed large-scale clustering algorithm, which employs a hybrid strategy of sampling and partitioning to derive representative points, has demonstrated significant potential in balancing efficiency and accuracy. The algorithm’s ability to construct a minimum spanning tree from the representative points and segment it based on local density results in rapid and precise clustering outcomes. The extensive evaluations conducted on both synthetic and real-world data sets have validated the effectiveness of the proposed methodology, exhibiting swift execution, high precision, and resilience to variations in sampling and partitioning parameters. Furthermore, the algorithm’s capability to operate without a predetermined number of clusters adds to its flexibility and practicality. These findings suggest that the presented approach represents a valuable contribution to the field of large-scale clustering, particularly for applications where both efficiency and accuracy are crucial.

## Data Availability

The datasets used and/or analysed during the current study are available from the corresponding author on reasonable request.
